# Generation of *TGFBI* knockout ABCG2+/ABCB5+ double-positive limbal epithelial stem cells by CRISPR/Cas9-mediated genome editing

**DOI:** 10.1371/journal.pone.0211864

**Published:** 2019-02-12

**Authors:** Eung Kweon Kim, Seunghyuk Kim, Yong-Sun Maeng

**Affiliations:** 1 Department of Ophthalmology, Corneal Dystrophy Research Institute, Yonsei University College of Medicine, Seoul, South Korea; 2 Institute of Vision Research, Severance Biomedical Science Institute, Yonsei University College of Medicine, Seoul, South Korea; 3 Department of Obstetrics and Gynecology, Institute of Women’s Life Medical Science, Yonsei University College of Medicine, Seoul, Republic of Korea; Cedars-Sinai Medical Center, UNITED STATES

## Abstract

Corneal dystrophy is an autosomal dominant disorder caused by mutations of the transforming growth factor β-induced (*TGFBI*) gene on chromosome 5q31.8. This disease is therefore ideally suited for gene therapy using genome-editing technology. Here, we isolated human limbal epithelial stem cells (ABCG2+/ABCB5+ double-positive LESCs) and established a TGFBI knockout using RNA-guided clustered regularly interspaced short palindromic repeats (CRISPR)/Cas9 genome editing. An LESC clone generated with a single-guide RNA (sgRNA) targeting exon 4 of the *TGFBI* gene was sequenced in order to identify potential genomic insertions and deletions near the Cas9/sgRNA-target sites. A detailed analysis of the differences between wild type LESCs and the single LESC clone modified by the *TGFBI*-targeting sgRNA revealed two distinct mutations, an 8 bp deletion and a 14 bp deletion flanked by a single point mutation. These mutations each lead to a frameshift missense mutation and generate premature stop codons downstream in exon 4. To validate the TGFBI knockout LESC clone, we used single cell culture to isolate four individual sub-clones, each of which was found to possess both mutations present in the parent clone, indicating that the population is homogenous. Furthermore, we confirmed that TGFBI protein expression is abolished in the TGFBI knockout LESC clone using western blot analysis. Collectively, our results suggest that genome editing of TGFBI in LESCs by CRISPR/Cas9 may be useful strategy to treat corneal dystrophy.

## Introduction

Corneal dystrophies are slowly progressive, symmetric, and unrelated to systemic or environmental factors [1]. Bilateral corneal deposits resulting from these conditions can cause photophobia, tearing, pain, and eventually reduce visual acuity [2]. The advent of genetic analysis has allowed the identification of transforming growth factor β-induced gene (TGFBI) mutations that are associated with specific corneal dystrophies. For example, p.Arg124Leu is found in Reis-Bücklers corneal dystrophy (RBCD), p.Arg555Gln leads to Thiel-Behnke corneal dystrophy (TBCD), p.Arg124Cys causes lattice corneal dystrophy type 1 (LCD1), p.Arg555Trp results in granular corneal dystrophy type 1 (GCD1), and p.Arg124His occurs in granular corneal dystrophy type 2 (GCD2) [1]. Overall, 57 mutations in the *TGFBI* gene have been associated with corneal dystrophies.

Based on published studies, p.Arg124His (GCD2) is the most frequently observed mutation in the Asian population, with the second most common mutation likely to be either p.Arg124Cys (LCD1) [3] or p.Arg555Trp (GCD1) [4]. A Japanese report, for example, found that the GCD2 mutation accounted for up to 72% of patients in their study population [3, 4]. Our group has identified 21 individuals that are homozygous for this mutation in Korea and calculated that heterozygotes are likely to account for 1 out of every 870 Korean people [5, 6]. China was distinct from other Asian countries in that the GCD1 mutation was most frequently identified in *TGFBI* corneal dystrophy patients, followed by the LCD1 and GCD2 mutations [7]. Further, in Western countries, LCD1 was most common genetic variant in this disease.

The corneal epithelium arises from, and is maintained by, limbal epithelial stem cells (LESCs) in the basal layer of the corneal limbus. These multiply slowly giving rise to transient amplifying cells (TACs), which migrate superficially while becoming more and more differentiated [8–10]. Limbal stem cell deficiency (LSCD) can arise for a number of reasons, including burn, injury, and infection. Due to a lack of corneal donor tissue and the decreased of graft survival after penetrating keratoplasty, stem cell therapies based on the autologous or homologous expansion of LESCs has been proposed in severe cases of LSCD [11].

LESCs are identified by expression of ΔNp63α along with a high nuclear to cytoplasmic ratio [12, 13]. ABCG2 (ATP binding cassette sub family G member 2) positivity detected in LESCs as well as several other cells exist in the suprabasal limbus and these markers used to identify the LESC population based on their staining ability in clusters of stem-like cells in the limbus [14, 15].

ABCB5 (ATP-binding cassette subfamily B member 5) is a regulator of limbal stem cell behavior and is required for corneal development [16]. ABCB5 was mainly expressed in basal layer cells of the mouse limbus. In human eyes, ABCB5^+^ cells were located in the basal layer of the limbus and co-expressed ΔNp63α− a known expressed in epithelial stem cells [16, 17], including human limbal stem cells[18, 19].

Recently, we isolated ABCG2^+^/ABCB5^+^ LESCs and confirmed differentiation of LESC into corneal epithelial cell [17]. The ABCG2+/ABCB5+ LESCs that we established displayed powerful stem cell activity, continuous growth, and high telomerase activity. Moreover, ABCG2+/ABCB5+ LESCs expressed the core transcription factors Oct4, Sox2, c-Myc, and Klf4, which are also expressed in multipotent stem cells [17]. These data indicate that the ABCG2+/ABCB5+ LESCs that we established have powerful stem cell activity and may be used to regenerate corneal epithelia. Based on these data, knock out of mutant TGFBIp in ABCG2+/ABCB5+ LESC from corneal dystrophy patients may be treatment strategy for corneal dystrophy patients.

Recently, an RNA-mediated adaptive immune system found in bacteria and archaea, known as clustered regularly interspaced short palindromic repeats (CRISPR) has been used to develop a revolutionary technology for gene editing in cells and organisms [20–25]. This CRISPR/Cas9 system uses the bacterial Cas9 protein, combined with a short single-guide RNA (sgRNA), which together can be used to generate targeted double-stranded breaks in the genomic DNA [26]. Additionally, cytoplasmic microinjections of *in vitro* transcribed mRNA combined with the CRISPR/Cas9 technology have been successfully used for genome modifications (correction of genetic disorders or disruption of the mutated gene) in cells, as well as in several types of mammalian embryos [27–30].

Because corneal dystrophy is commonly caused by dominant mutations in the *TGFBI* gene, we hypothesize that this disease is suited for gene therapy with genome-editing technology. Here, we present the use of CRISPR/Cas9 gene editing to knock out endogenous human *TGFBI* expression at the genome level in ABCG2+/ABCB5+ double-positive LESCs, resulting in the establishment of a *TGFBI* gene knockout clone. Our results suggest that genome editing of *TGFBI* in human LESCs by CRISPR/Cas9 may be useful strategy to treat corneal dystrophy.

## Materials and methods

### ABCG2+/ABCB5+ double-positive LESCs culture

Human corneal tissue was harvested from healthy corneas from the eye bank after penetrating or lamellar keratoplasty. The age, gender and health of donors are listed in Table 1. Donor confidentiality was maintained in accordance with the Declaration of Helsinki and was approved by the Severance Hospital IRB Committee (CR04124), Yonsei University. ABCG2+/ABCB5+ double-positive LESCs were isolated as previously described.[17] In brief, Fresh corneoscleral tissue was cut into four similar segments in a 60-mm culture dish containing HBSS (HBSS: Hank’s balanced salt solution), and each segment was digested with 15 mg/mL Dispase II (Roche, Rotkreuz, Switzerland) in SHEM (CELLnTEC Advanced Cell Systems AG, Bern, Switzerland) with 100 mM sorbitol (Sigma-Aldrich, St Louis, MO) at 4°C for 18 hours. Under a dissecting microscope, an already loose limbal epithelial sheet was separated by inserting and sliding a noncutting flat stainless-steel spatula into a plane between the limbal epithelium and the stroma. Isolated limbal epithelial cells were seeded on specific matrix [5% matrigel (BD Biosciences, Bedford, MA) and 0.05 mg/ml human fibronectin (Sigma-Aldrich, St Louis, MO)] coating plate and cultured with CnT-20 medium (CELLnTEC Advanced Cell Systems AG, Bern, Switzerland). After 3 days, limbal epithelial cells were cultured with 10% Serum DMEM (Invitrogen, Carlsbad, CA) medium and changed at every 2 days. After 8–10 days, highly proliferative cell colonies were washed with PBS two times and treated with 1 mL Accutase (Sigma-Aldrich, St Louis, MO). Single cells were seeded onto plates coated with a matrix of Matrigel and fibronectin and cultured in DMEM containing 10% FBS. The highly proliferative cells that attached to the new plate were designated limbal epithelial stem cells (LESCs). After 48 hours, The LESCs were treated with Accutase and sorted by FACS analysis using ABCG2+ antibodies (Abcam, Cambridge, MA) and ABCB5+ antibodies (Thermo fisher scientific, Rockford, IL). The ABCG2+/ABCB5+ double-positive cells were cultured using mass culture methods and were named ABCG2+/ABCB5+ double-positive LESCs.

**Table 1:** Human donor information.

	Male (N = 3)	Female (N = 3)
Mean age	37.6±11.02	31±7.8
Systolic blood pressure (mmHg)	120±9.8	115±10.5
Diastolic blood pressure(mmHg)	75±12.5	73±11.3
BMI (kg/m2)	19.9±3.4	20.1±5.9
Hemoglobin (g/dl)	14.5±0.7	14.0±0.4
Glucose level(mg/dl)	81±5.9	80±4.7
Creatinine (mg/dl)	0.9±0.11	0.8±0.12
AST(IU/L)	21±2.2	20±2.3
ALT(IU/L)	20±2.4	19±2.1
γGTP(IU/L)	45±3.5	23±2.7
Proteinuria	Normal	Normal

### LESC transfection

Transfections were performed in 24-well cell culture plates with confluencies of approximately 60–70% using 1.5 μg of the *TGFBI*-targeting sgRNA (TCAGCTGTACACGGACCGCACGG) plasmid, 1 μg of the Cas9 plasmid, and 10 μl of Lipofectamine Reagent (Invitrogen, Carlsbad, CA), or negative control using only 1 μg of the Cas9 plasmid, and 10 μl of Lipofectamine Reagent, according to the manufacturer’s instructions. Transfected cells were cultured for 24 h; these were then harvested, diluted in cell culture medium to approximately 1 cell/100 μl, and re-plated in 96-well cell culture plates. Once individual colonies were apparent in 66 of two 96-well plate, these were cultured in separate wells of 24-well plates and, subsequently, further expanded in 6-well and 60 mm plates until cell numbers were sufficient for genomic DNA extraction and western blot analyses.

### Design of sgRNA/Cas9 vectors

Basically, we focused on granular corneal dystrophy type 2 (GCD2). Substitution of arginine for histidine at codon 124 (p.Arg124His) is associated with this disease. Therefore, we chose exon 4 as the target of sgRNA in both NHEJ to knockout and HDR to correct the mutation. Custom-designed CRISPR/Cas9 vectors, targeting one specific region of exon 4 of the *TGFBI* gene, were obtained from Toolgen (South Korea). First, four sgRNA-target sites were selected by an algorithm, and one of the sgRNA-target sites was selected by an algorithm that suggests sites with minimal risk to generate off-target effects and by mismatched sensitive nuclease assay (T7E1 assay). Selected sgRNA-target sites have the best cutting efficiency and fewer predicted off-target sites. Single-guide (sg) RNAs targeting the *TGFBI* gene were under the regulation of a U6 promoter, whereas expression of the Cas9 enzyme was driven by a cytomegalovirus promoter.

### DNA mismatch-specific (T7E1) endonuclease assay

LESCs transfected with the *TGFBI*-targeting sgRNA plasmid and the Cas9 plasmid were harvested after 3 days of growth, and genomic DNA was extracted using the QIAamp DNA Blood Kit (QIAGEN, Valencia, CA). A region of the *TGFBI* gene exon 4 was amplified with genomic DNA-specific primers (forward primer, 5’-GTTCACGTAGACAGGCATTTGA-3’; reverse primer, 5’-GCCTTTTCTAAGGGGTTAAGGA-3’). Homoduplex PCR products were then denatured and rehybridized using step-down annealing conditions to generate homo- and heteroduplexes, and the duplex mixture was treated with T7E1 nuclease for 1 h at 37°C (New England Biolabs, Ipswich, MA). The reaction was stopped using 1.5 μl of 0.25 M EDTA, and the products were analyzed on a 2% agarose gel.

### Western blot

Cells were grown to near confluency in 60 mm plates. Growth medium was then removed, and the cells were rinsed twice with phosphate buffered saline (PBS) prior to lysis with a radioimmunoprecipitation assay (RIPA) buffer, supplemented with phosphatase and protease inhibitors. Insoluble cell debris was removed by centrifugation for 15 min at 13,000 rpm and 4°C, and the protein levels were adjusted based on a bicinchoninic acid colorimetric (BCA) assay. Equal amounts of protein were separated using 10% sodium dodecyl sulfate-polyacrylamide gel electrophoresis (SDS-PAGE) and transferred to a polyvinyl difluoride (PVDF) membranes. These membranes were blocked in tris-buffered saline with Tween 20 (TBST) containing 5% skim milk and incubated with the primary anti-hTGFBI antibody (1:3,000, Abfrontier, Seoul, South Korea) overnight at 4°C. Blot membranes were then incubated with anti-rabbit secondary antibody (1:5,000, Thermo scientific, Rockford, IL, USA) in 5% skim milk in TBST, and the immunoreactive bands were visualized with a chemiluminescent reagent as recommended by Amersham Biosciences, Inc.

### Statistical analysis

Each experiment was repeated at least three times in triplicate. Data were performed using GraphPad Prism 5 software. Measurements are presented as means ± SE. Comparisons between two groups were analyzed by Student’s *t*-test. Multiple comparisons were performed by one-way ANOVA followed by either Dunnett’s or Tukey’s post hoc test.

## Results

### CRISPR/Cas9-mediated modification of TGFBI in LESCs

**Fig 1:**
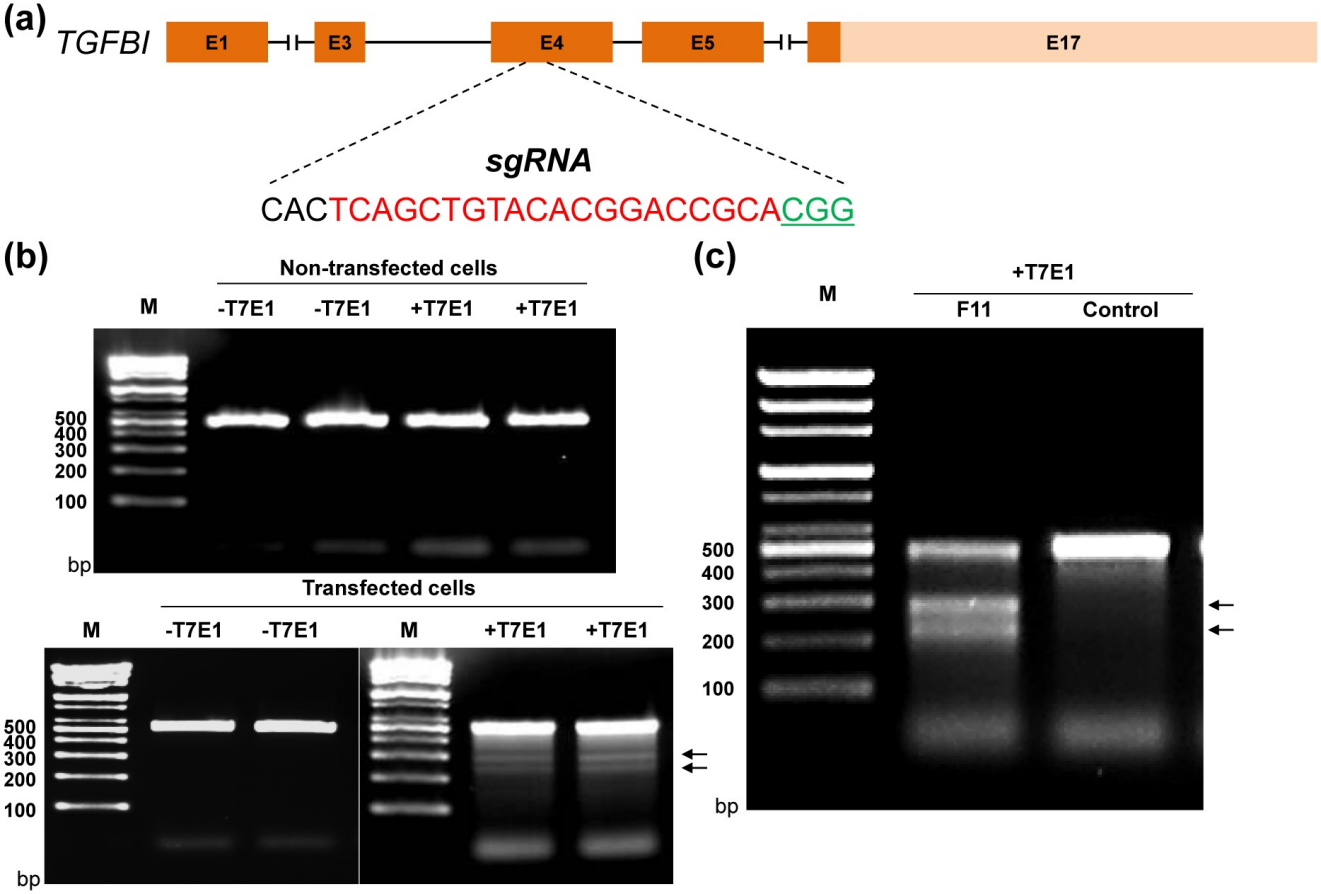
Evaluation of the sgRNA/Cas9-mediated TGFBI modifications in LESCs. (a) Schematic diagram of the *TGFBI* partial protein coding region and the locus targeted by the sgRNA/Cas9 complex. The sgRNA-targeting site is presented in red, and the PAM sequence is shown in green and underlined. Amino acid sequence was presented under nucleotide sequence. (b) Targeting efficiency test for the genome editing constructs. Genomic PCR (gPCR) products spanning *TGFBI* exon 4 were amplified from a heterogeneous population of LESCs that were transfected with or without the *TGFBI*-targeting sgRNA and Cas9 expression plasmids. Denaturation of these products, followed by rehybridization and treatment with T7E1 nuclease for 1 h at 37°C results in bands of ~280 bp and ~220 bp, indicated with arrows. This is consistent with the predicted cleavage sizes of 284 bp and 227 bp. M, marker. (c) Screening of single clones from LESCs transfected with *TGFBI*-targeting sgRNA and Cas9 expression plasmids. Single cells were cultured in 96-well plates until colonies were visible, with media changes every 2 days. These were dissociated and moved first to 24-well plates and then to 6-well plates, and gPCR products spanning exon 4 of *TGFBI* were amplified from individual single cell clones. The gPCR products were denatured, rehybridized, and treated with T7E1 nuclease for 1 h at 37°C, and the products were analyzed on a 2% agarose gel. LESC clone F11 was found to be T7E1 nuclease positive, producing cleavage products of ~280 bp and ~220 bp. Arrows indicate bands resulting from T7E1 nuclease cleavage.

We first identified an appropriate target site in *TGFBI* for gene editing by screening 20 bp target sequences and 3 bp protospacer associated motif (PAM) sequences in exon 4 of the human *TGFBI* gene that was filtered by the Toolgen company to minimize off-target cross-reactivity. From a list of several candidates, we chose a target sequence spanning bases 354–373 of the human *TGFBI* cDNA. On the minus strand, these nucleotides are positioned immediately 5’ to the trinucleotide PAM sequence “CGG” (Fig 1a). Once identified, an oligo pair containing the guide sequence was cloned into the pRGEN-U6 vector to produce the pRGEN-U6-TGFBI plasmid; we also obtained the pRGEN-Cas9-CMV expression vector, which expresses the Cas9 enzyme, from the Toolgen company.

We chose to use primary human ABCG2+/ABCB5+ double-positive LESCs as the model system for generating *TGFBI* knockout cells using the CRISPR/Cas9 system. Novel limbus- derived, highly proliferative ABCG2+/ABCB5+ double-positive LESCs were established in our previous research.[17] To test the effectiveness of the *TGFBI*-targeting sgRNA at triggering Cas9-mediated gene editing at the target site, LESCs were transiently co-transfected with the pRGEN-U6-TGFBI sgRNA plasmid and the pRGEN-Cas9-CMV vector. This resulted in the generation of a heterogeneous total population of edited and non-edited cells. At 72 h post-transfection, we extracted total genomic DNA from this population and PCR-amplified a genomic region containing the target site in exon 4 of *TGFBI*. We then denatured and re-annealed the PCR amplicons in a thermal cycler to generate heteroduplex pairs and subjected these rehybridized products to digestion with T7E1 nuclease. The T7E1 enzyme selectively recognizes and cleaves mismatched bracket sites and heteroduplexes harboring indels [31]. Since CRISPR/Cas9 complexes trigger double-stranded breaks and imperfect non-homologous end joining (NHEJ) near the PAM [21], we predicted that T7E1 digestion of mismatches in the target site should generate DNA fragments of ~227 bp and ~284 bp in size. As shown in Fig 1b, the results of this T7E1 efficiency assay suggested that the *TGFBI*-targeting sgRNA was functional.

We therefore attempted to isolate single LESC clones targeted by the *TGFBI* sgRNA by co-transfecting LESCs with the sgRNA and Cas9 protein expression plasmids and culturing individual clones derived from a single cell each in 96-well plates. These clones were allowed to grow until colonies formed, with fresh media provided every 2 days. Single clones were then dissociated and moved to 24-well plates and, subsequently, to 6-well plates. Genomic DNA was isolated from each clone, and PCR reactions were performed as described above. The products were rehybridized, treated with T7E1 nuclease for 1 h at 37°C, and analyzed on a 2% agarose gel. We found that the F11 single LESC clone was T7E1 nuclease positive, and produced a PCR product that was cleaved to yield products of ~280 and ~220 bp (Fig 1c).

**Fig 2:**
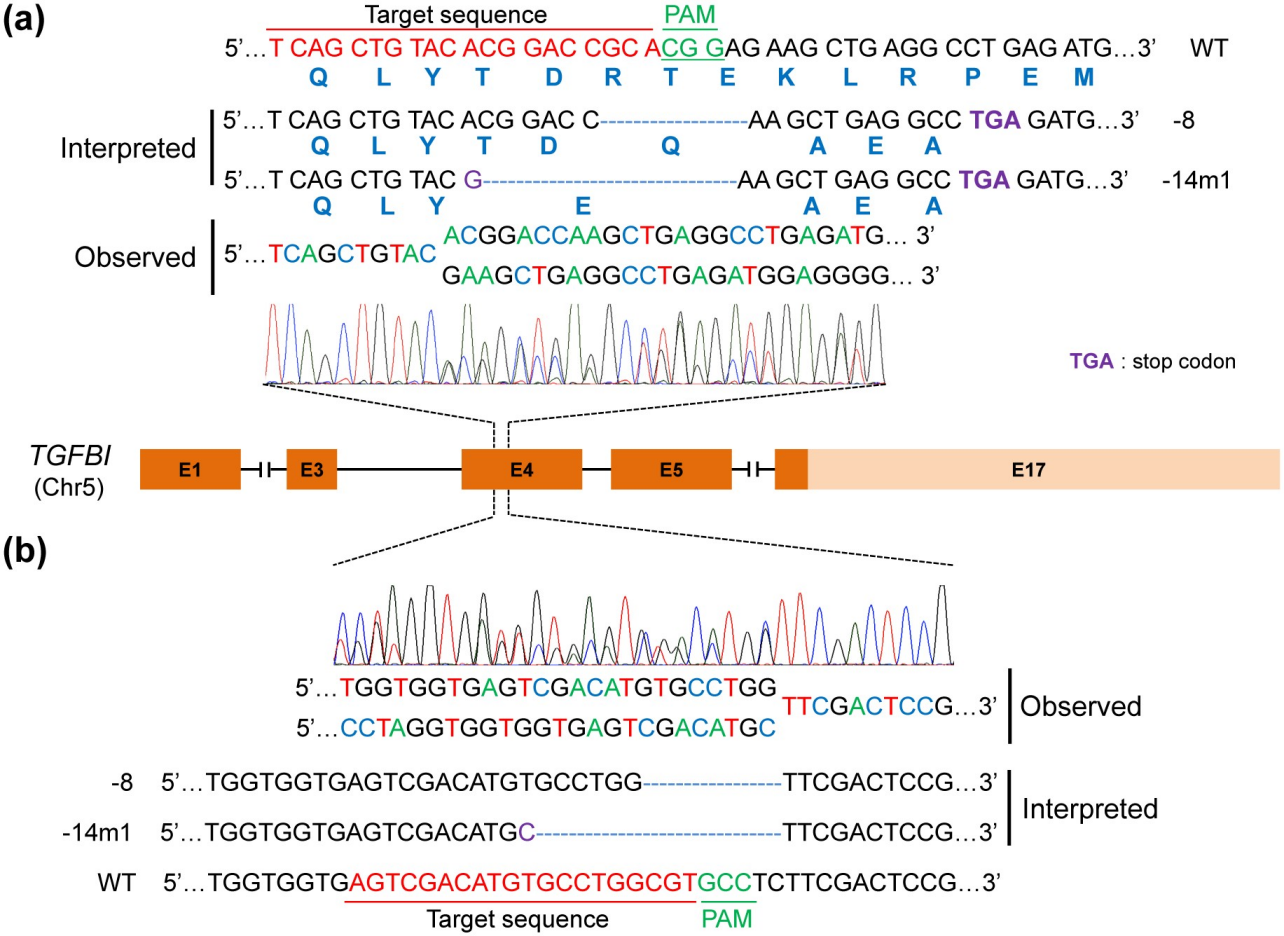
Insertion/Deletion (indel) analysis for the F11 single LESC clone. Sanger sequencing of gPCR products spanning exon 4 of *TGFBI* and containing the *TGFBI* sgRNA target site to identify indels in the F11 single LESC clone. (a) Results of the forward sequencing reaction. The wild-type (WT) sequence was identified in control LESCs, and two distinct mutant sequences, an 8 bp deletion and a 14 bp deletion flanked by a single point mutation, were identified in the F11 clone. These mutations each lead to a frameshift missense mutation and the generation of a premature stop codon downstream of the target site in exon 4. The 20-nt sgRNA target site, 3-nt PAM sequence, and stop codon site are highlighted in red, green, and purple, respectively. Deletions (-) or nucleotide mutation (m) are shown to the right of each allele. (b) Results of the reverse sequencing reaction. The WT sequence was identified in control LESCs, and the two mutant sequences described in (a, b) were identified in the F11 single LESC clone.

We next compared the indel sequences found near the *TGFBI* sgRNA target site in the F11 single LESC clone to the corresponding region in wild-type LESCs using Sanger sequencing, and observed overlapping peaks in the sequencing chromatographs (Fig 2). From these data, we determined that two different mutations were present, an 8 bp deletion and a 14 bp deletion flanked by one point mutation, and these were present in both the forward (Fig 2a) and reverse (Fig 2b) sequencing reactions. We did not observe a single wild type *TGFBI* sequence in either the forward or reverse sequencing results from the F11 single LESC clone. Intriguingly, these mutations both lead to a frameshift missense mutation and the formation of a premature stop codon downstream in exon 4.

### Validation of TGFBI knockout LESCs

**Fig 3:**
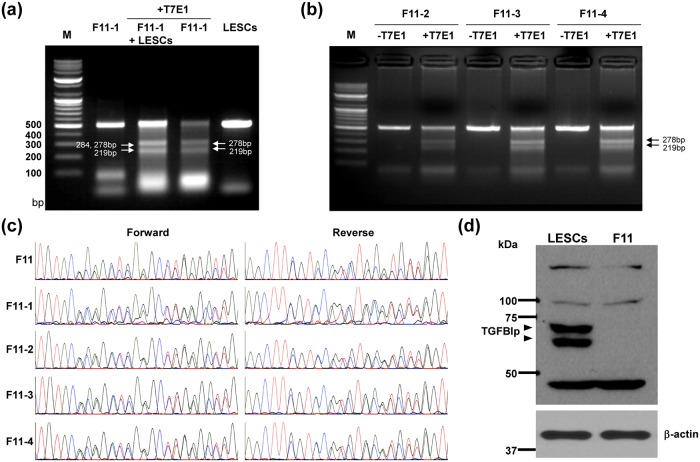
Validation of TGFBI knockout LESCs. (a, b) The F11 LESC clone was single-cell cultured in 96-well plates, and individual subclones were allowed to reach 80% confluency, with media changes every 2 days. Genomic DNA was extracted from four F11 subclones, F11-1, -2, -3, and -4, and gPCR products spanning exon 4 of *TGFBI* were amplified. These were denatured, rehybridized, and treated with T7E1 nuclease for 1 h at 37°C, both individually and when mixed with the gPCR products from WT LESCs, and the resulting fragments were analyzed on a 2% agarose gel. Cleavage products of 278 bp and 219 bp were present after T7E1 nuclease treatment of products from individual F11 subclones, and fragments of 284 bp, 278 bp, and 219 bp were generated by T7E1 nuclease treatment of mixed reactions with gPCR products from WT LESCs. M, marker. (c) Results of forward and reverse sequencing reactions of gPCR products spanning *TGFBI* exon 4 from single subclones of the F11 LESC clone. Both mutant sequences found in the F11 parent were identified in all single cell subclones. (d) TGFBI protein levels in cell lysates (35 μg) from WT LESCs and the F11 single LESC clone were determined by Western blot analysis using an anti-TGFBI primary antibody and an anti-β-actin antibody, as a loading control. Arrowheads indicate the position of the band corresponding to the TGFBI protein. Intact TGFBI protein is absent in the F11 clone.

Because our F11 single LESC clone generated by the *TGFBI*-targeting sgRNA contained two distinct types of mutation (i.e., an 8 bp deletion and a 14 bp deletion with an adjacent point mutation, Fig 2a and 2b), we wondered whether this clone has mono-allelic CRISPR-mediated 8bp deletion or 14bp deletion with single point mutation or rather, if some cells has bi-allelic 8bp deletion and others has bi-allelic 14bp deletion with single point mutation. To answer this question, we subcloned single cells from the F11 single LESC clone in 96-well plates and obtained four single cell subclones, which we termed F11-1, -2, -3, and -4. We then extracted genomic DNA from these subclones and amplified PCR products spanning exon 4 of *TGFBI*. PCR products from each single cell clone, as well as a mixture of the PCR product obtained from wild type LESCs and a single cell clone, were rehybridized, treated with T7E1 nuclease, and analyzed on a 2% agarose gel. We predicted that if the F11 single LESC clone has a mono-allelic CRISPR-mediated 8bp deletion or 14bp deletion with single point mutation, the T7E1 assay would be positive for both the single cell clones and the mixed reaction, containing PCR products from wild type LESCs and the single cell clones. Conversely, if some cells has bi-allelic 8bp deletion and others has bi-allelic 14bp deletion with single point mutation, some of which contain the 8 bp deletion and some of which contain the 14 bp deletion/single point mutation, the T7E1 assay would be positive only for the mixed reactions containing PCR products from wild type LESCs and the single cell clones. We found that both the PCR products from the single cell clones and the mixed PCR products from wild type LESCs and the single cell clones were cleaved by T7E1 nuclease (Fig 3a and 3b), suggesting that the F11 clone has mono-allelic CRISPR-mediated 8bp deletion or 14bp deletion with single point mutation.

To further validate our hypothesis, we analyzed sequences near the *TGFBI* sgRNA target site in the F11 single LESC clone and in the four single cell subclones, F11-1, -2, -3, and -4, using Sanger sequencing (Fig 3c). We found that all cells possessed the same sequences and mutations, that is, the 8 bp deletion and the 14 bp deletion flanked by a single point mutation, near the *TGFBI* sgRNA target site. Therefore, we conclude that the F11 single LESC clone is composed of homogeneous population of cells that contain two distinct *TGFBI* loci.

The predicted consequence of both the 8 bp deletion and the 14 bp deletion flanked by a single point mutation in the F11 clone is a frameshift, which generates four new amino acids and a premature stop codon downstream of alanine 127 or alanine 125, respectively (Fig 2). Thus, this LESC clone lacks the intact wild-type allele, and all of the genomic DNA sequences that were identified contained deletions that create nonfunctional gene products. To confirm the loss of TGFBI protein (TGFBIp) expression in the knockout cells, we performed a western blot analysis on total protein lysates from normal LESCs and the F11 single LESC clone using the human TGFBI polyclonal antibody and the β-actin antibody, as a loading control (Fig 3d). Our data show the complete lack of detectable TGFBIp expression in the F11 single LESC clone, thus confirming the generation of a *TGFBI* knockout cell line using the CRISPR/Cas9 system.

In Fig 3D, TGFBIp showed two bands. Based on our previous studies, TGFBIp showed one or two bands (68kDa and additional smaller size bands) by western blotting, dependent on the cell conditions or cell type [32–35]. However, we are unsure why TGFBIp showed one or two bands. There are two possibilities. One is that the cells express two isoforms of TGFBIp depending on cell conditions or cell type. Another is that the cells express an enzyme that modifies TGFBIp depending on the cell condition or cell type. Our current results suggest that the cells express an enzyme that modifies TGFBIp under different cell conditions. (unpublished data). Therefore, TGFBIp showed one or two bands on the western blot. We are currently verifying this hypothesis.

## Discussion

Transforming growth factor beta-induced gene (TGFBI; BIGH3; bigh3) encodes transforming growth factor beta-induced protein (TGFBIp), composed of 683 amino acid residues. TGFBIp is known to expressed ubiquitously in various organs including pancreas, heart, skin, liver [36], bone [37], endometrium [38], kidney [39], tendon [40], and blood plasma [41].

TGFBIp is thought to play important roles in physio-pathologic responses by mediating cell adhesion [36, 42–44] migration [42, 43], proliferation and differentiation [44]. TGFBIp mediate cell adhesion and/or spreading through integrins α1β1, α3β1, ανβ3, ανβ5, α6β4, and αmβ2 [43, 45–49] and also associated with metastasis and suppression of malignant tumors [42, 50, 51]. Recently, our report suggested that TGFBIp increases migration, adhesion and differentiation of lymphatic endothelial cells so that inhibition of TGFBIp expression resulted in reduction of tumor lymphangiogenesis. These effects finally inhibited the metastasis of TGFBIp-producing tumors [34, 35]. We also reported that TGFBIp increases the migration and adhesion of endothelial progenitor cells through integrins α4 and α5 [52]. In the cornea, TGFBIp is expressed mainly in the epithelium [53], and up-regulated significantly during wound healing of the cornea, and increased the mucins expression [54, 55].

In cornea, mutation of *TGFBI* gene induces 5q31- linked autosomal dominant corneal dystrophies [56]. These diseases are characterized by accumulation of deposits in the cornea, often culminates in blindness due to the accumulation of protein deposits in the cornea. Munier et al. recognized the relationships between TGFBI mutations and specific corneal dystrophies [1]: p.Arg124Leu is found in Reis-Bücklers corneal dystrophy (RBCD), p.Arg555Gln leads to Thiel-Behnke corneal dystrophy (TBCD), p.Arg124Cys causes lattice corneal dystrophy type 1 (LCD1), p.Arg555Trp results in granular corneal dystrophy type 1 (GCD1), and p.Arg124His occurs in granular corneal dystrophy type 2 (GCD2) [1]. Overall, 57 mutations in the *TGFBI* gene have been associated with corneal dystrophies.

Immunohistological studies showed that wild-type TGFBIp exists mainly in the extracellular space of corneal epithelial cells [53], while mutant TGFBIp is abundant in the pathologic deposits in TGFBIp-related corneal dystrophies [53]. TGFBIp presents in both a free soluble form and a covalently bound state [57]. The soluble TGFBIp may serve a regulatory function, while the bound state TGFBIp may exhibit as anchors for cells in the ECM. Therefore, interaction between TGFBIp and collagen is important for understanding the pathobiology of *TGFBI*-linked corneal dystrophies. However, the role of wild- and mutant-type TGFBIp in corneal epithelial cells is largely unknown, despite its clear expression in the cornea.

Over the years, our understanding of the pathogenesis of *TGFBI*-related corneal dystrophies has advanced significantly, but much remains to be learned. Currently, several surgical techniques have tried to treating visually significant deposits in corneal dystrophy patients. However, recent efforts have focused on the development of topical medications that might prevent the deposition of mutant TGFBIp and/or dissolve existing deposits. Gene therapy using RNA interference (RNAi), which can silence a disease-associated mutant allele, has been investigated to treat diseases such as corneal dystrophy. In using of RNAi for gene suppression, there are two commonly used methods: small interfering RNAs (siRNAs), and short hairpin siRNAs (shRNAs). Yuan *et al*. generated a shRNA, which was able to reduce the levels of TGFBIp in a transformed HEK 293 cell line transfected with a TGFBI expression plasmid [58]. Courtney *et al*. further developed an allele-specific siRNA targeting the TGFBI-Arg124Cys LCD1 variant, and this was able to reduce both the mutant *TGFBI* expression and amyloid aggregate formation *in vitro* [59]. Because TGFBIp plays multiple physiological roles, however, the non-specific nature of this siRNAs raises concerns regarding the safety of their clinical application.

The CRISPR/Cas9 system has, in recent years, been extensively applied for gene editing in various organisms [22, 28, 60, 61]. This system uses the Cas9 protein combined with a sgRNA, which together, promote targeted double-stranded breaks in the genomic DNA [26]. Genome modification by CRISPR/Cas9 has dramatically accelerated in the genomic editing field and has successfully been employed to correct the Duchenne muscular dystrophy of mouse [29]. Ultimately, gene therapy with tools such as CRISPR/Cas9 system may provide an effective treatment strategy to repair the gene sequences mutated in *TGFBI*-related corneal dystrophies.

LESCs are located in the basal layer of the corneal limbus [62, 63] and are responsible for the repair [64, 65] and maintenance of the corneal surface [66, 67]. Disease and injury can lead to a deficiency of LESCs, resulted the corneas becoming opaque, vascularized, and inflamed. Cultured LESC therapy was first described in 1997 [68], and LESCs cultured from either patients or donors have been used to successfully treat LSCD.

Corneal dystrophy is commonly caused by dominant mutations in the *TGFBI* gene, and thus, we hypothesize that this is a disease ideally suited for gene therapy with genome editing technology. Here, we co-transfected human ABCG2+/ABCB5+ double-positive LESCs with plasmids expressing the *TGFBI*-targeting sgRNA (pRGEN-U6-TGFBI) and the Cas9 protein (pRGEN-Cas9-CMV) and isolated a single *TGFBI* gene knockout LESC clone. This clone (F11) was shown to comprise a homogeneous population of cells, each of which contains two distinct *TGFBI* loci, one with an 8 bp deletion and another that has a 14 bp deletion flanked by a single point mutation. Intriguingly, both these mutations lead to a frame-shift missense mutation and generate premature stop codons downstream in exon 4. In addition, we performed whole-genome sequencing to analyze the CRISPR/Cas9-system based off-target effects. However, F11 single clone cells showed no off-target effects on the genome. Therefore, we suggest that the selected sgRNA is safe for use in the treatment of stem cells from patient's. The successful knockout of *TGFBI* was confirmed by western blot, which showed the complete lack of detectable TGFBIp expression in the F11 single LESC clone. These findings confirm the generation of a *TGFBI* knockout LESC cell line using the CRISPR/Cas9 system, and to our knowledge, our study is the first report describing the successful targeting of *TGFBI* using this technology.

Collectively, our results suggest that CRISPR/Cas9 genome editing targeting the *TGFBI* gene in human ABCG2+/ABCB5+ double-positive LESCs may be applied therapeutically in corneal dystrophy patients. Specifically, we predict that autologous transplantation of LESCs containing either a *TGFBI* gene knockout or a corrected *TGFBI* allele represents a feasible treatment strategy for corneal dystrophy patients. We also anticipate that the cell clones described in this report will be useful to the research community studying the pathogenesis of corneal dystrophy diseases. Importantly, the CRISPR/Cas9-mediated genome editing described here can now easily be adapted for the generation of additional *TGFBI* knockout clones in other cell lines, and future studies will be aimed at correcting the *TGFBI* gene mutations present in corneal dystrophy using advanced CRISPR/Cas9 systems. When combined with more conventional *in vitro* cell manipulation approaches, these new tools may not only facilitate the identification of both the cellular function of TGFBI and signaling pathways critical for corneal dystrophy diseases but also provide new treatment options for patients suffering from this disease.

## Conclusions

Genome editing of *TGFBI* in human ABCG2+/ABCB5+ double-positive LESCs by CRISPR/Cas9 may be useful strategy to treat corneal dystrophy, and these new tools may not only facilitate the identification of both the cellular function of TGFBI and signaling pathways critical for corneal dystrophy diseases but also provide new treatment options for patients suffering from this disease.

## References

[1] Munier FL, Korvatska E, Djemai A, Le Paslier D, Zografos L, Pescia G, et al Kerato-epithelin mutations in four 5q31-linked corneal dystrophies. Nat Genet. 1997;15(3):247–51. 10.1038/ng0397-247 . 9054935

[2] Klintworth GK. Advances in the molecular genetics of corneal dystrophies. Am J Ophthalmol. 1999;128(6):747–54. . 10612512 10.1016/s0002-9394(99)00358-x

[3] Fujiki K, Nakayasu K, Kanai A. Corneal dystrophies in Japan. J Hum Genet. 2001;46(8):431–5. 10.1007/s100380170041 . 11501939

[4] Mashima Y, Yamamoto S, Inoue Y, Yamada M, Konishi M, Watanabe H, et al Association of autosomal dominantly inherited corneal dystrophies with BIGH3 gene mutations in Japan. Am J Ophthalmol. 2000;130(4):516–7. . 11024425 10.1016/s0002-9394(00)00571-7

[5] Lee JH, Cristol SM, Kim WC, Chung ES, Tchah H, Kim MS, et al Prevalence of granular corneal dystrophy type 2 (Avellino corneal dystrophy) in the Korean population. Ophthalmic Epidemiol. 2010;17(3):160–5. 10.3109/09286581003624939 . 20455845

[6] Cho KJ, Mok JW, Na KS, Rho CR, Byun YS, Hwang HS, et al TGFBI gene mutations in a Korean population with corneal dystrophy. Mol Vis. 2012;18:2012–21. . 22876129 PMC3413419

[7] Yang J, Han X, Huang D, Yu L, Zhu Y, Tong Y, et al Analysis of TGFBI gene mutations in Chinese patients with corneal dystrophies and review of the literature. Mol Vis. 2010;16:1186–93. . 20664689 PMC2901189

[8] Tseng SC. Concept and application of limbal stem cells. Eye (Lond). 1989;3 (Pt 2):141–57. 10.1038/eye.1989.22 . 2695347

[9] Kinoshita S, Friend J, Thoft RA. Sex chromatin of donor corneal epithelium in rabbits. Invest Ophthalmol Vis Sci. 1981;21(3):434–41. . 7024181

[10] Sun TT, Tseng SC, Lavker RM. Location of corneal epithelial stem cells. Nature. 2010;463(7284):E10–1; discussion E1. 10.1038/nature08805 . 20182462

[11] Rama P, Matuska S, Paganoni G, Spinelli A, De Luca M, Pellegrini G. Limbal stem-cell therapy and long-term corneal regeneration. N Engl J Med. 2010;363(2):147–55. 10.1056/NEJMoa0905955 . 20573916

[12] Arpitha P, Prajna NV, Srinivasan M, Muthukkaruppan V. High expression of p63 combined with a large N/C ratio defines a subset of human limbal epithelial cells: implications on epithelial stem cells. Invest Ophthalmol Vis Sci. 2005;46(10):3631–6. 10.1167/iovs.05-0343 . 16186343

[13] Pellegrini G, Rama P, Matuska S, Lambiase A, Bonini S, Pocobelli A, et al Biological parameters determining the clinical outcome of autologous cultures of limbal stem cells. Regen Med. 2013;8(5):553–67. 10.2217/rme.13.43 . 23725042

[14] Mariappan I, Maddileti S, Savy S, Tiwari S, Gaddipati S, Fatima A, et al In vitro culture and expansion of human limbal epithelial cells. Nat Protoc. 2010;5(8):1470–9. 10.1038/nprot.2010.115 . 20671730

[15] Osei-Bempong C, Figueiredo FC, Lako M. The limbal epithelium of the eye—a review of limbal stem cell biology, disease and treatment. Bioessays. 2013;35(3):211–9. 10.1002/bies.201200086 . 23129317

[16] Ksander BR, Kolovou PE, Wilson BJ, Saab KR, Guo Q, Ma J, et al ABCB5 is a limbal stem cell gene required for corneal development and repair. Nature. 2014;511(7509):353–7. 10.1038/nature13426 . 25030174 PMC4246512

[17] Kim EK, Lee GH, Lee B, Maeng YS. Establishment of Novel Limbus-Derived, Highly Proliferative ABCG2(+)/ABCB5(+) Limbal Epithelial Stem Cell Cultures. Stem Cells Int. 2017;2017:7678637 10.1155/2017/7678637 . 29230251 PMC5694571

[18] Liu L, Nielsen FM, Emmersen J, Bath C, Ostergaard Hjortdal J, Riis S, et al Pigmentation Is Associated with Stemness Hierarchy of Progenitor Cells Within Cultured Limbal Epithelial Cells. Stem Cells. 2018;36(9):1411–20. 10.1002/stem.2857 . 29781179

[19] Bojic S, Hallam D, Alcada N, Ghareeb A, Queen R, Pervinder S, et al CD200 Expression Marks a Population of Quiescent Limbal Epithelial Stem Cells with Holoclone Forming Ability. Stem Cells. 2018;36(11):1723–35. 10.1002/stem.2903 . 30157305

[20] Jinek M, Chylinski K, Fonfara I, Hauer M, Doudna JA, Charpentier E. A programmable dual-RNA-guided DNA endonuclease in adaptive bacterial immunity. Science. 2012;337(6096):816–21. 10.1126/science.1225829 . 22745249 PMC6286148

[21] Cong L, Ran FA, Cox D, Lin S, Barretto R, Habib N, et al Multiplex genome engineering using CRISPR/Cas systems. Science. 2013;339(6121):819–23. 10.1126/science.1231143 . 23287718 PMC3795411

[22] Li D, Qiu Z, Shao Y, Chen Y, Guan Y, Liu M, et al Heritable gene targeting in the mouse and rat using a CRISPR-Cas system. Nat Biotechnol. 2013;31(8):681–3. 10.1038/nbt.2661 . 23929336

[23] Mali P, Yang L, Esvelt KM, Aach J, Guell M, DiCarlo JE, et al RNA-guided human genome engineering via Cas9. Science. 2013;339(6121):823–6. 10.1126/science.1232033 . 23287722 PMC3712628

[24] Wang H, Yang H, Shivalila CS, Dawlaty MM, Cheng AW, Zhang F, et al One-step generation of mice carrying mutations in multiple genes by CRISPR/Cas-mediated genome engineering. Cell. 2013;153(4):910–8. 10.1016/j.cell.2013.04.025 . 23643243 PMC3969854

[25] Doudna JA, Charpentier E. Genome editing. The new frontier of genome engineering with CRISPR-Cas9. Science. 2014;346(6213):1258096 10.1126/science.1258096 . 25430774

[26] Garneau JE, Dupuis ME, Villion M, Romero DA, Barrangou R, Boyaval P, et al The CRISPR/Cas bacterial immune system cleaves bacteriophage and plasmid DNA. Nature. 2010;468(7320):67–71. 10.1038/nature09523 . 21048762

[27] Schwank G, Koo BK, Sasselli V, Dekkers JF, Heo I, Demircan T, et al Functional repair of CFTR by CRISPR/Cas9 in intestinal stem cell organoids of cystic fibrosis patients. Cell Stem Cell. 2013;13(6):653–8. 10.1016/j.stem.2013.11.002 . 24315439

[28] Crispo M, Mulet AP, Tesson L, Barrera N, Cuadro F, dos Santos-Neto PC, et al Efficient Generation of Myostatin Knock-Out Sheep Using CRISPR/Cas9 Technology and Microinjection into Zygotes. PLoS One. 2015;10(8):e0136690 10.1371/journal.pone.0136690 . 26305800 PMC4549068

[29] Long C, McAnally JR, Shelton JM, Mireault AA, Bassel-Duby R, Olson EN. Prevention of muscular dystrophy in mice by CRISPR/Cas9-mediated editing of germline DNA. Science. 2014;345(6201):1184–8. 10.1126/science.1254445 . 25123483 PMC4398027

[30] Ding Q, Strong A, Patel KM, Ng SL, Gosis BS, Regan SN, et al Permanent alteration of PCSK9 with in vivo CRISPR-Cas9 genome editing. Circ Res. 2014;115(5):488–92. 10.1161/CIRCRESAHA.115.304351 . 24916110 PMC4134749

[31] Cho SW, Kim S, Kim JM, Kim JS. Targeted genome engineering in human cells with the Cas9 RNA-guided endonuclease. Nature biotechnology. 2013;31(3):230–2. 10.1038/nbt.2507 . 23360966

[32] Choi SI, Jin JY, Maeng YS, Kim TI, Kim EK. TGF-beta regulates TGFBIp expression in corneal fibroblasts via miR-21, miR-181a, and Smad signaling. Biochem Biophys Res Commun. 2016;472(1):150–5. 10.1016/j.bbrc.2016.02.086 . 26915797

[33] Choi SI, Maeng YS, Kim TI, Lee Y, Kim YS, Kim EK. Lysosomal trafficking of TGFBIp via caveolae-mediated endocytosis. PLoS One. 2015;10(4):e0119561 10.1371/journal.pone.0119561 . 25853243 PMC4390356

[34] Maeng YS, Lee R, Lee B, Choi SI, Kim EK. Lithium inhibits tumor lymphangiogenesis and metastasis through the inhibition of TGFBIp expression in cancer cells. Sci Rep. 2016;6:20739 10.1038/srep20739 . 26857144 PMC4746585

[35] Maeng YS, Aguilar B, Choi SI, Kim EK. Inhibition of TGFBIp expression reduces lymphangiogenesis and tumor metastasis. Oncogene. 2016;35(2):196–205. 10.1038/onc.2015.73 . 25772247

[36] Skonier J, Neubauer M, Madisen L, Bennett K, Plowman GD, Purchio AF. cDNA cloning and sequence analysis of beta ig-h3, a novel gene induced in a human adenocarcinoma cell line after treatment with transforming growth factor-beta. DNA Cell Biol. 1992;11(7):511–22. 10.1089/dna.1992.11.511 . 1388724

[37] Kitahama S, Gibson MA, Hatzinikolas G, Hay S, Kuliwaba JL, Evdokiou A, et al Expression of fibrillins and other microfibril-associated proteins in human bone and osteoblast-like cells. Bone. 2000;27(1):61–7. . 10865210 10.1016/s8756-3282(00)00292-1

[38] Carson DD, Lagow E, Thathiah A, Al-Shami R, Farach-Carson MC, Vernon M, et al Changes in gene expression during the early to mid-luteal (receptive phase) transition in human endometrium detected by high-density microarray screening. Mol Hum Reprod. 2002;8(9):871–9. . 12200466 10.1093/molehr/8.9.871

[39] Lee SH, Bae JS, Park SH, Lee BH, Park RW, Choi JY, et al Expression of TGF-beta-induced matrix protein betaig-h3 is up-regulated in the diabetic rat kidney and human proximal tubular epithelial cells treated with high glucose. Kidney Int. 2003;64(3):1012–21. 10.1046/j.1523-1755.2003.00158.x . 12911551

[40] Ferguson JW, Thoma BS, Mikesh MF, Kramer RH, Bennett KL, Purchio A, et al The extracellular matrix protein betaIG-H3 is expressed at myotendinous junctions and supports muscle cell adhesion. Cell Tissue Res. 2003;313(1):93–105. 10.1007/s00441-003-0743-z . 12838408

[41] Klintworth GK, Valnickova Z, Enghild JJ. Accumulation of beta ig-h3 gene product in corneas with granular dystrophy. Am J Pathol. 1998;152(3):743–8. . 9502416 PMC1858399

[42] Nam JO, Jeong HW, Lee BH, Park RW, Kim IS. Regulation of tumor angiogenesis by fastatin, the fourth FAS1 domain of betaig-h3, via alphavbeta3 integrin. Cancer Res. 2005;65(10):4153–61. 10.1158/0008-5472.CAN-04-2705 . 15899806

[43] Park SW, Bae JS, Kim KS, Park SH, Lee BH, Choi JY, et al Beta ig-h3 promotes renal proximal tubular epithelial cell adhesion, migration and proliferation through the interaction with alpha3beta1 integrin. Exp Mol Med. 2004;36(3):211–9. 10.1038/emm.2004.29 . 15272232

[44] Park SY, Jung MY, Kim IS. Stabilin-2 mediates homophilic cell-cell interactions via its FAS1 domains. FEBS Lett. 2009;583(8):1375–80. 10.1016/j.febslet.2009.03.046 . 19328203

[45] Kim HJ, Kim IS. Transforming growth factor-beta-induced gene product, as a novel ligand of integrin alphaMbeta2, promotes monocytes adhesion, migration and chemotaxis. Int J Biochem Cell Biol. 2008;40(5):991–1004. 10.1016/j.biocel.2007.11.001 . 18083624

[46] Kim JE, Jeong HW, Nam JO, Lee BH, Choi JY, Park RW, et al Identification of motifs in the fasciclin domains of the transforming growth factor-beta-induced matrix protein betaig-h3 that interact with the alphavbeta5 integrin. J Biol Chem. 2002;277(48):46159–65. 10.1074/jbc.M207055200 . 12270930

[47] Kim MO, Yun SJ, Kim IS, Sohn S, Lee EH. Transforming growth factor-beta-inducible gene-h3 (beta(ig)-h3) promotes cell adhesion of human astrocytoma cells in vitro: implication of alpha6beta4 integrin. Neurosci Lett. 2003;336(2):93–6. . 12499048 10.1016/s0304-3940(02)01260-0

[48] Nam EJ, Sa KH, You DW, Cho JH, Seo JS, Han SW, et al Up-regulated transforming growth factor beta-inducible gene h3 in rheumatoid arthritis mediates adhesion and migration of synoviocytes through alpha v beta3 integrin: Regulation by cytokines. Arthritis Rheum. 2006;54(9):2734–44. 10.1002/art.22076 . 16947382

[49] Ohno S, Noshiro M, Makihira S, Kawamoto T, Shen M, Yan W, et al RGD-CAP ((beta)ig-h3) enhances the spreading of chondrocytes and fibroblasts via integrin alpha(1)beta(1). Biochim Biophys Acta. 1999;1451(1):196–205. . 10446401 10.1016/s0167-4889(99)00093-2

[50] Fan Y, Shen B, Tan M, Mu X, Qin Y, Zhang F, et al TGF-beta-induced upregulation of malat1 promotes bladder cancer metastasis by associating with suz12. Clin Cancer Res. 2014;20(6):1531–41. 10.1158/1078-0432.CCR-13-1455 . 24449823

[51] Massague J. TGFbeta in Cancer. Cell. 2008;134(2):215–30. 10.1016/j.cell.2008.07.001 . 18662538 PMC3512574

[52] Maeng YS, Choi YJ, Kim EK. TGFBIp regulates differentiation of EPC (CD133(+) C-kit(+) Lin(-) cells) to EC through activation of the Notch signaling pathway. Stem Cells. 2015;33(6):2052–62. 10.1002/stem.2003 . 25786978

[53] Escribano J, Hernando N, Ghosh S, Crabb J, Coca-Prados M. cDNA from human ocular ciliary epithelium homologous to beta ig-h3 is preferentially expressed as an extracellular protein in the corneal epithelium. J Cell Physiol. 1994;160(3):511–21. 10.1002/jcp.1041600314 . 8077289

[54] Rawe IM, Zhan Q, Burrows R, Bennett K, Cintron C. Beta-ig. Molecular cloning and in situ hybridization in corneal tissues. Invest Ophthalmol Vis Sci. 1997;38(5):893–900. . 9112985

[55] Maeng YS, Lee GH, Lee B, Choi SI, Kim TI, Kim EK. Role of TGFBIp in Wound Healing and Mucin Expression in Corneal Epithelial Cells. Yonsei Med J. 2017;58(2):423–31. 10.3349/ymj.2017.58.2.423 . 28120575 PMC5290024

[56] Gibson MA, Kumaratilake JS, Cleary EG. Immunohistochemical and ultrastructural localization of MP78/70 (betaig-h3) in extracellular matrix of developing and mature bovine tissues. J Histochem Cytochem. 1997;45(12):1683–96. 10.1177/002215549704501212 . 9389772

[57] Hirano K, Klintworth GK, Zhan Q, Bennett K, Cintron C. Beta ig-h3 is synthesized by corneal epithelium and perhaps endotheliumin Fuchs' dystrophic corneas. Curr Eye Res. 1996;15(9):965–72. . 8921218 10.3109/02713689609017642

[58] Yuan C, Zins EJ, Clark AF, Huang AJ. Suppression of keratoepithelin and myocilin by small interfering RNAs (siRNA) in vitro. Mol Vis. 2007;13:2083–95. . 18079684

[59] Courtney DG, Atkinson SD, Moore JE, Maurizi E, Serafini C, Pellegrini G, et al Development of allele-specific gene-silencing siRNAs for TGFBI Arg124Cys in lattice corneal dystrophy type I. Invest Ophthalmol Vis Sci. 2014;55(2):977–85. 10.1167/iovs.13-13279 . 24425855

[60] Ott de Bruin L, Yang W, Capuder K, Lee YN, Antolini M, Meyers R, et al Rapid generation of novel models of RAG1 deficiency by CRISPR/Cas9-induced mutagenesis in murine zygotes. Oncotarget. 2016 10.18632/oncotarget.7341 . 26887046 PMC4914335

[61] Yuan L, Sui T, Chen M, Deng J, Huang Y, Zeng J, et al CRISPR/Cas9-mediated GJA8 knockout in rabbits recapitulates human congenital cataracts. Sci Rep. 2016;6:22024 10.1038/srep22024 . 26912477 PMC4766569

[62] Schermer A, Galvin S, Sun TT. Differentiation-related expression of a major 64K corneal keratin in vivo and in culture suggests limbal location of corneal epithelial stem cells. J Cell Biol. 1986;103(1):49–62. . 2424919 10.1083/jcb.103.1.49 PMC2113783

[63] Schlotzer-Schrehardt U, Kruse FE. Identification and characterization of limbal stem cells. Exp Eye Res. 2005;81(3):247–64. 10.1016/j.exer.2005.02.016 . 16051216

[64] Notara M, Alatza A, Gilfillan J, Harris AR, Levis HJ, Schrader S, et al In sickness and in health: Corneal epithelial stem cell biology, pathology and therapy. Exp Eye Res. 2010;90(2):188–95. 10.1016/j.exer.2009.09.023 . 19840786

[65] Xie HT, Chen SY, Li GG, Tseng SC. Isolation and expansion of human limbal stromal niche cells. Invest Ophthalmol Vis Sci. 2012;53(1):279–86. 10.1167/iovs.11-8441 . 22167096 PMC3292364

[66] Chen JJ, Tseng SC. Abnormal corneal epithelial wound healing in partial-thickness removal of limbal epithelium. Invest Ophthalmol Vis Sci. 1991;32(8):2219–33. . 1712763

[67] Mort RL, Ramaesh T, Kleinjan DA, Morley SD, West JD. Mosaic analysis of stem cell function and wound healing in the mouse corneal epithelium. BMC Dev Biol. 2009;9:4 10.1186/1471-213X-9-4 . 19128502 PMC2639382

[68] Pellegrini G, Traverso CE, Franzi AT, Zingirian M, Cancedda R, De Luca M. Long-term restoration of damaged corneal surfaces with autologous cultivated corneal epithelium. Lancet. 1997;349(9057):990–3. 10.1016/S0140-6736(96)11188-0 . 9100626

